# Tooth agenesis and orofacial clefting: genetic brothers in arms?

**DOI:** 10.1007/s00439-016-1733-z

**Published:** 2016-10-03

**Authors:** M. Phan, F. Conte, K. D. Khandelwal, C. W. Ockeloen, T. Bartzela, T. Kleefstra, H. van Bokhoven, M. Rubini, H. Zhou, C. E. L. Carels

**Affiliations:** 1 Department of Orthodontics and Craniofacial Biology, Radboud University Medical Center, Nijmegen, The Netherlands; 2 Department of Human Genetics, Radboud University Medical Center, Radboud Institute for Molecular Life Sciences, Nijmegen, The Netherlands; 3 Department of Molecular Developmental Biology, Faculty of Science, Radboud Institute for Molecular Life Sciences, Radboud University, Nijmegen, The Netherlands; 4 Department of Orthodontics, Dentofacial Orthopedics and Pedodontics, Center for Dental and Craniofacial Sciences, Charité-Universitätsmedizin Berlin, Berlin, Germany; 5 Department of Biomedical and Specialty Surgical Sciences, Medical Genetic Unit, University of Ferrara, Ferrara, Italy; 6 Department of Oral Health Sciences, Faculty of Medicine, KU Leuven and University Hospitals KU Leuven, Kapucijnenvoer, 7, 3000 Leuven, Belgium

## Abstract

Tooth agenesis and orofacial clefts represent the most common developmental anomalies and their co-occurrence is often reported in patients as well in animal models. The aim of the present systematic review is to thoroughly investigate the current literature (PubMed, EMBASE) to identify the genes and genomic loci contributing to syndromic or non-syndromic co-occurrence of tooth agenesis and orofacial clefts, to gain insight into the molecular mechanisms underlying their dual involvement in the development of teeth and facial primordia. Altogether, 84 articles including phenotype and genotype description provided 9 genomic loci and 26 gene candidates underlying the co-occurrence of the two congenital defects: *MSX1*, *PAX9*, *IRF6*, *TP63, KMT2D*, *KDM6A*, *SATB2*, *TBX22*, *TGFα*, *TGFβ3*, *TGFβR1*, *TGFβR2*, *FGF8*, *FGFR1*, *KISS1R*, *WNT3*, *WNT5A*, *CDH1*, *CHD7*, *AXIN2*, *TWIST1*, *BCOR*, *OFD1*, *PTCH1*, *PITX2*, and *PVRL1*. The molecular pathways, cellular functions, tissue-specific expression and disease association were investigated using publicly accessible databases (EntrezGene, UniProt, OMIM). The Gene Ontology terms of the biological processes mediated by the candidate genes were used to cluster them using the *GOTermMapper* (Lewis-Sigler Institute, Princeton University), speculating on six super-clusters: (a) anatomical development, (b) cell division, growth and motility, (c) cell metabolism and catabolism, (d) cell transport, (e) cell structure organization and (f) organ/system-specific processes. This review aims to increase the knowledge on the mechanisms underlying the co-occurrence of tooth agenesis and orofacial clefts, to pave the way for improving targeted (prenatal) molecular diagnosis and finally to reflect on therapeutic or ultimately preventive strategies for these disabling conditions in the future.

**Electronic supplementary material:**

The online version of this article (doi:10.1007/s00439-016-1733-z) contains supplementary material, which is available to authorized users.

## Introduction

Developmental tooth abnormalities, including mild and more severe forms of tooth agenesis (TA), have often been reported in patients affected with orofacial clefts (OFCs) (Ranta 1986; Aspinall et al. 2014). We recently observed that the same genes whose mutations were shown to cause TA, such as *MSX1* and *PAX9* (Seo et al. 2013), often also contain SNPs as genetic risk factors for OFCs.

Both, TA and OFCs represent two of the most common developmental orofacial birth defects. While hypodontia—the agenesis of 1–5 teeth (excluding agenesis of third molars)—is highly prevalent (more than 5 % in some populations), severe TA—oligodontia with agenesis of 6 teeth or more (excluding agenesis of third molars)—has been estimated to affect 1 individual in 1000 worldwide (Rakhshan and Rakhshan 2015; Polder et al. 2004). For OFCs, the overall prevalence has been estimated as 1 in 700–1000 live births (Mossey and Catilla 2003). These statistics, however, do not convey the considerable variation across studies depending on the severity of the phenotype; the study design, the cohort ethnicity and the geographical location also affect the prevalence (Khalaf et al. 2014; Murthy and Bhaskar 2009; Vastardis et al. 1996). Both conditions lead to significant life-long complications that require extensive multidisciplinary treatments, and represent severe psychosocial and economic burdens for their families and for society (Mossey et al. 2009).

**Fig. 1:**
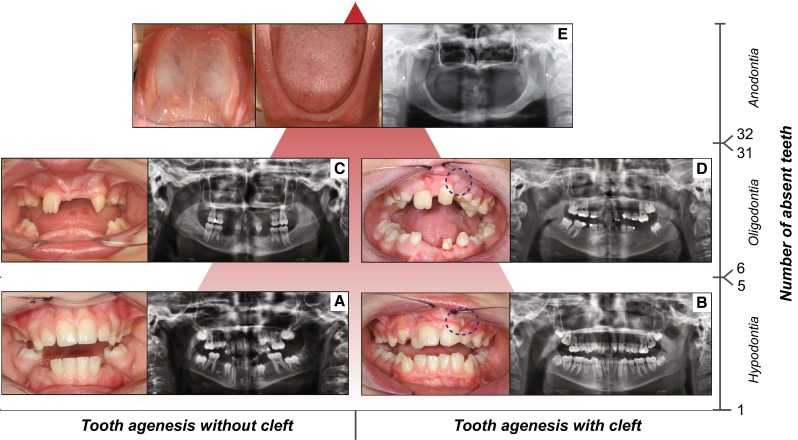
Forms of tooth agenesis. *Panel* of tooth agenesis (TA) forms in the permanent dentition, listed according to the number of absent teeth. Frontal intraoral pictures and orthopantograms (OPTs) of two adult patients affected with hypodontia, **a** without cleft and **b** with cleft (repaired cleft lip involving the alveolar ridge, marked by *dashed blue circle*), respectively. Frontal intraoral pictures and OPTs of two adult patients affected by oligodontia, **c** without cleft and **d** with cleft (repaired cleft lip and palate involving the alveolar ridge, marked by *dashed blue circle*), respectively. **e** Internal intraoral pictures (maxillary dental arch, *left*; mandibular dental arch, *right*) and OPT of an adult patient affected by complete anodontia, without orofacial clefts (copyright: Wang et al. 2013). *X*-axis: presence or absence of orofacial cleft in combination with TA. *Y*-axis: number of absent teeth (hypodontia, 1–5 missing teeth; oligodontia 6–31 missing teeth; anodontia, 32 missing teeth)

**Fig. 2:**
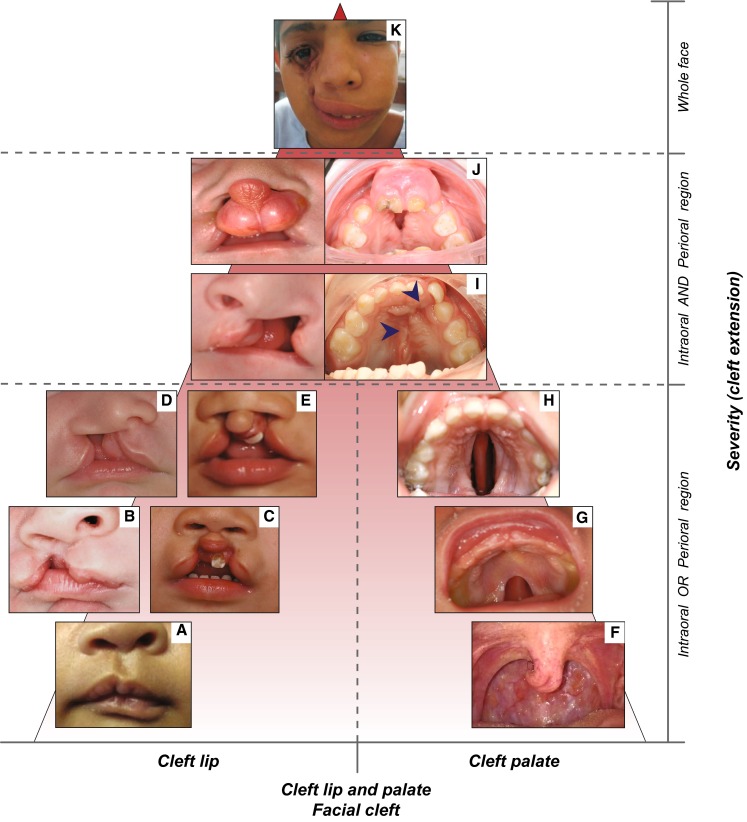
Forms of orofacial clefts. *Panel* of orofacial cleft forms, listed according to the severity based on the cleft extension and orofacial regions affected. Cleft lip types (frontal views): microform (**a**) (copyright: Cleft lip—A comprehensive review. Shkoukani et al., Front Pediatr. 2013); unilateral incomplete cleft lip (**b**); bilateral incomplete cleft lip (**c**); unilateral complete cleft lip (**d**); bilateral complete cleft lip (**e**). Cleft palate types (occlusal views): bifid uvula (**f**); cleft of the soft palate (**g**); cleft of hard and soft palate (**h**). Unilateral cleft lip and palate (**i**): frontal view of the patient in childhood and occlusal view of the same patient in adulthood, where the cleft palate has been repaired (surgical scars marked with *blue arrows*). Bilateral cleft lip and palate (**j**): frontal view of the patient in childhood, with protruding vermilion, and occlusal view of the same patient in adulthood, where the cleft palate is still partially open. Unilateral facial cleft extending from the oral region till the eye (K) (copyright: Garg and Goyal 2009). *X*-axis: type of orofacial cleft. *Y*-axis: severity based on the cleft extension (intraoral region, perioral region, whole face)

Based on the number of missing teeth, TA is conventionally divided into three forms: hypodontia, oligodontia and anodontia (Klein et al. 2013). Hypodontia (HD) is used for one to five missing teeth, whereas oligodontia (OD) is used for six or more missing teeth (Fig. 1). Anodontia (AD) is the most severe condition with complete lack of tooth development in the deciduous and permanent dentition (Fig. 1). As the third molars are missing in up to 20 % of the populations worldwide, making it a very common finding, these teeth are excluded from the classification (Vastardis et al. 1996; Graber 1978). Based on the severity and the anatomical regions involved, OFCs are also classified into different phenotypic categories ranging from microforms to rare complete overt facial clefts, i.e., oblique facial cleft, where the gap may extend to the nose, the cheeks, the eyes, the ears till the forehead (Fig. 2). The three main OFC phenotypes are represented by cleft lip (CL), cleft palate (CP) and cleft lip and palate (CLP), which can be uni- or bilateral (Fig. 2).

In CL, the nasal and lip primordia fail to fuse resulting in a gap of the upper lip and the disruption of the orbicularis oris muscle, with a variable degree of severity ranging from microforms, i.e., forme fruste CL, to complete unilateral or bilateral clefting. CP is characterized by either a submucosal or an overt cleft in the anterior hard palate or posterior soft palate, with variable disorientation of palatal muscles, arising from the fusion failure of lateral palatal shelves. The mildest form of soft CP involves only the uvula, while in the most severe cases the cleft extends through soft and secondary hard palate. CLP is a combination of the previously described phenotypes, usually divided into two classes: incomplete CLP (a.k.a. cleft lip and alveolus) when the upper lip, alveolar ridge and part of the hard palate (primary palate) are affected, or complete CLP, when the cleft develops along the entire mouth length from the nostrils to the uvula. Despite their common features, CLP, CP and CL emerge from the disruption of distinct morphogenetic processes at different stages of embryological development (Shkoukani et al. 2013).

Both TA and OFCs can occur as isolated conditions without any other recognizable anomaly (non-syndromic forms) or associated with structural abnormalities of other anatomical regions (syndromic forms) (Cobourne 2004; Klein et al. 2013). Over 80 syndromes include TA among their typical features, especially HD, while over 275 syndromes include at least one of the different subtypes of OFCs (Klein et al. 2013; Leslie and Marazita 2013). Interestingly, syndromic forms of TA and OFCs may arise within the same syndromes: this is the case for van der Woude syndrome (VWS) (OMIM# 119300), which includes OFCs with dental anomalies and lip fistulas (Kondo et al. 2002).

In a recent comprehensive study based on the largest international cohort of individuals with OFC investigated so far (Howe et al. 2015), it has been shown that a wide spectrum of dental anomalies, characterized by alteration in tooth number, size, shape, timing of formation and eruption, is more frequently detected in individuals with OFC than in the population without these birth defects, although this evidence is restricted to the upper jaw. The prevalence of TA in and outside the cleft area, as well as its location in the upper versus lower jaw, has been reported to be significantly higher in patients with OFC compared to individuals without a cleft (Shapira et al. 1999; Aspinall et al. 2014). TA has been described to occur approximately three times more frequently on the cleft than on the non-cleft side (Ranta 1972), and its severity increases with the OFC phenotype severity (Ranta 1986). The cause of the co-occurrence of these dental abnormalities and OFCs has also been debated. According to Howe et al. (2015), the dental features may result from local mechanical circumstances at the time of the cleft formation or from conditions of blood supply during early postnatal surgical interventions.

In their geometric morphometric study in a *Neo/Null* and *Neo/Wt* mouse model, Green et al. (2015) show that the facial/nasal prominences can fail to fuse due to their misalignment as a result of decreased mesenchymal growth. Failure of tooth germ development can also be caused by mutations in genes which regulate mesenchymal cell proliferation (like *MSX1*), fitting the common genetic origin hypothesis (Eerens et al. 2001). Such gene variants could therefore—besides causing TA—also increase the risk for OFC development, if a proper alignment of the midfacial prominences is not achieved in time. Moreover, the absence of developing tooth germ structures (like thickened dental laminas in the growing palatal processes) could itself also underlie the subtle volumetric shape changes contributing to the failure of optimal geometric alignment of the approaching orofacial prominences. In the Online Mendelian Inheritance in Man (OMIM) database an overall large genetic heterogeneity for selective TA (STHAG) is described, but so far only STHAG type 1 (OMIM# 106600) includes the annotation ‘with or without orofacial cleft’, which draws back to a heterozygous mutation affecting *MSX1* (Table 1, Supplementary Table 4) (van den Boogaard et al. 2000). Combined TA and OFC phenotypes in humans have, however, been also shown to result from rare variants of *IRF6* and *TP63*, both in syndromic and non-syndromic cases (Celli et al. 1999; McGrath et al. 2001; Brunner et al. 2002a, b; Kondo et al. 2002).

**Table 1:** Genes contributing to the non-syndromic co-occurrence OFCs and TA

Gene	Study	No. of cases	Type of OFC	Type of TA	TA location	Comments^a^	References
AXIN2	Case–control study	500	OFCs	TA	Unclear	Borderline association for CDH1 and AXIN2 markers	Letra et al. (2009)
CDH1	Case–control study	500	OFCs	TA	Unclear	Borderline association for CDH1 and AXIN2 markers	Letra et al. (2009)
IRF6	Population-based case–control study	108	OFCs	TA	Unclear	Markers of two genes investigated: IRF6 and TGFα	Letra et al. (2012)
	Population-based case–control study	9	OFCs	TA	Outside	Significant association of IRF6 SNP (rs642961) in homo-/heterozygous patients with isolated OFCs and TA. Determination of TA outside the cleft	Krasone et al. (2014)
MSX1	Family-based study	3	OFCs	TA	Inside and outside	Three members from same Dutch family. TA location: 18, 28, 38, 48, 15, 25, 35, 45, 14, 24, 22	van den Boogaard et al. (2000)
	Case–control study	57	OFCs	HD	Inside and outside	Unrelated patients. Significant markers on MSX1 and TGFB3. HD located outside cleft area for 36/57 patients	Slayton et al. (2003)
	Case–control study	19	OFCs	TA	Inside and outside	Unrelated patients. TA location: 15, 25, 35, 45, 13, 23, 33, 43, 31, 41	Modesto et al. (2006)
	Family-based study	2	CL	TA	Outside	One family with four affected members (only two analyzed). TA location: 18, 28, 38, 48, 17, 27, 37, 47, 15, 25, 35, 45, 14, 24, 12, 22, 31, 41	Liang et al. (2012)
	Population-based case–control study	126	OFCs	TA	Inside and outside	Significant association for MSX1 and PAX9 markers	Seo et al. (2013)
PAX9	Family-based study	2	OFCs	HD	Outside	Family showing dominant hypodontia	Das et al. (2003)
	Population-based case–control study	126	OFCs	TA	Inside and outside	Significant association for MSX1 and PAX9 markers	Seo et al. (2013)
TGFα	Population-based case–control study	108	OFCs	TA	Unclear	Markers of two genes investigated: IRF6 and TGFα	Letra et al. (2012)
TGFβ3	Case–control study	57	OFCs	HD	Inside and outside	Unrelated patients. Significant association for MSX1 and TGFB3 markers. HD located outside the cleft area for 36/57 patients	Slayton et al. (2003)

*OFCs* orofacial clefts, *CL/P* cleft lip with or without cleft palate, *CL* cleft lip, *TA* tooth agenesis, *HD* hypodontia, *OD* oligodontia

^a^When available, the missing teeth are indicated in the comments column with the official enumeration

The present study aims to systematically review the literature to provide a comprehensive panel of genes and loci reported to be associated to the co-occurrence of TA and OFCs in patients (syndromic and non-syndromic cases), including supporting evidence in animal models when available. This will not only increase the knowledge on the genetic risk factors and mechanisms underlying the co-occurrence of TA and OFCs, but will also pave the way to improve (prenatal) targeted diagnosis.

## Materials and methods

The literature search was systematically performed using two publicly available literature databases, PubMed (http://www.ncbi.nlm.nih.gov/pubmed) and EMBASE (https://ovidsp.tx.ovid.com/sp-3.17.0a/ovidweb.cgi), in August 2015. In each database, three separate searches were performed based on search terms belonging to three broad topics—genetics, orofacial clefts and tooth agenesis (Supplementary Table 1)—to avoid the risk of overlooking interesting articles. The individual searches were carried out using free text search combined with subject headings (Supplementary Table 1). In each database, the articles resulting from the individual searches were then overlapped to highlight only those containing terms from the three fields of interest in their abstract and title. Next, the final lists of overlapping articles from PubMed and EMBASE were both exported into EndNote X7 (*Thomson Reuters,* http://endnote.com), where the duplicates were removed and the article texts were retrieved.

In the first selection phase, the non-English language studies were excluded as well as the conference and meeting reports. Subsequently, the remaining articles were entirely screened and hence selected according to the inclusion criteria. In principle, the articles were included when describing evidence of genes or genetic loci—in human or in animal models—whose disruption may cause orofacial clefts (OFCs), specifically CL, CP or CL/P, and tooth agenesis (TA), including AD, OD or HD (especially located outside the cleft area), with or without other phenotypes. The evidence that leads to the inclusion of articles was based on phenotyping using clinical examination, X-rays, or histology in case of animal experiments, and on genotyping such as polymerase chain reaction and genome-wide association studies. The lack of molecular diagnosis, the absence of OFC or TA or the unclear phenotype description was reason enough to exclude an article. The authors M.P. and F.C. of this review first carried out the content-based selection of the articles individually while the disagreements about the study eligibility were solved by discussion and further careful check of the published data. In case both first authors found uncertainty in classifying an article, the authors of that article were contacted to ask for further clarifications before deciding on its inclusion or exclusion.

The molecular pathways, cellular functions, tissue-specific expression and disease association of the candidate genes collected from the included articles were investigated using publicly accessible databases, such as EntrezGene (www.ncbi.nlm.nih.gov/entrez/query.fcgi?db=gene), UniProt (www.uniprot.org/) and OMIM (http://www.omim.org/), highlighting the aspects that further support the hypothesis of association between the genes and the co-occurrence of OFCs and TA. In addition, the Gene Ontology terms indicating the biological processes mediated by these candidate genes were used to cluster them using the GO tool names *GOTermMapper* (Lewis-Sigler Institute for Integrative Genomics, Princeton University, http://go.princeton.edu/cgi-bin/GOTermMapper) based on the *map2slim* script, part of the GO Perl package (Boyle et al. 2004; Harris et al. 2004). This tool maps the granular GO annotations for each gene to a set of broad, high-level GO parent terms (GO-slim terms), allowing to bin the genes into general categories, which can eventually be summarized in even broader super-clusters.

Apart from genes, genomic loci were also collected: for each locus, the genomic coordinates were defined using UCSC Genome Browser (https://genome.ucsc.edu/index.html) and the encompassed genes (RefSeq genes) were retrieved with Table Browser, setting *GRCh38/hg38* as the human genome assembly.

## Results

### Inclusion and exclusion of articles in our study and dataflow chart

**Fig. 3:**
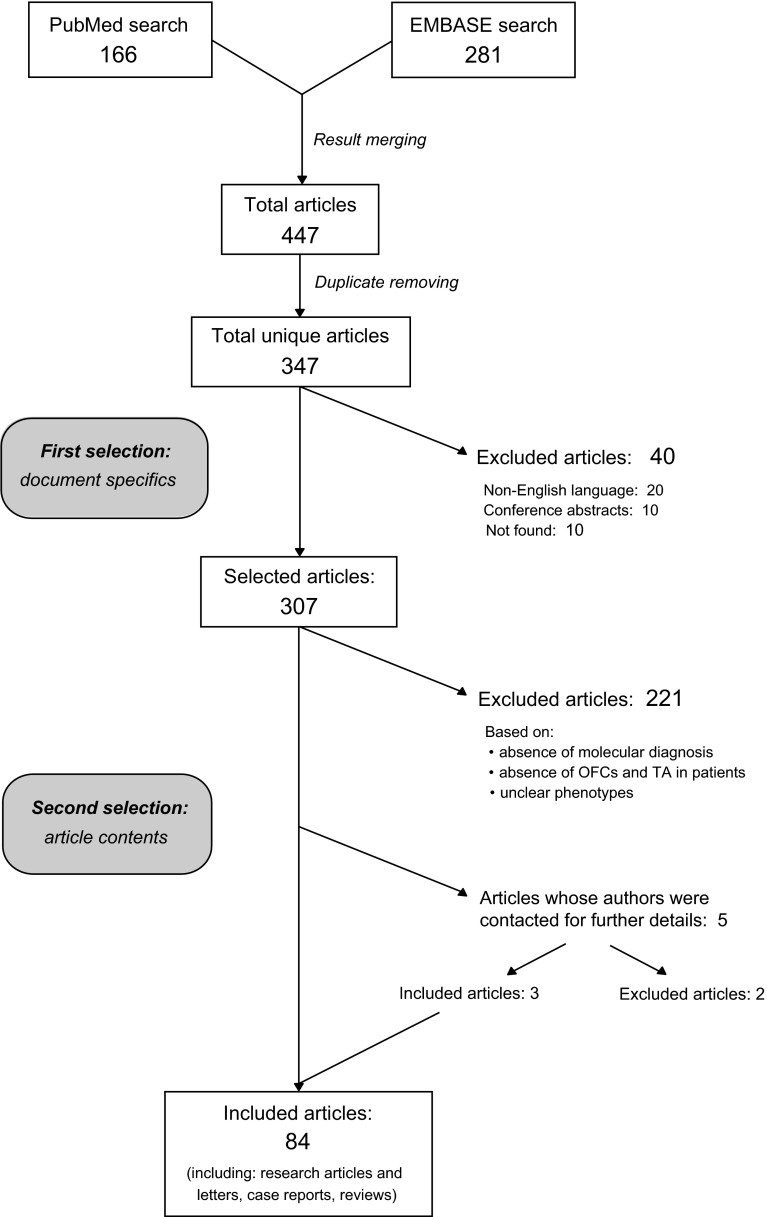
Search flowchart. The literature search was performed using PubMed, which provided 166 articles, and EMBASE, which provided 281 articles, combining to a total of 447 articles. After the removal of duplicates (100), the selection process was carried out in two steps. In the first selection, the references where screened based on the document specifics: non-English articles (20), conference reports (10) and not available articles (10) were removed. The second selection of the remaining 307 articles was based on the contents, considering the molecular diagnosis and the combined phenotypes (TA and OFCs) present in patients and animal models, excluding 221 articles. For five articles the authors were contacted, and three of them were subsequently included. The final number of selected articles was 84, including research articles, case reports, research letters and reviews

Our systematic search of the literature initially yielded 347 unique articles, of which 263 had to be excluded due to incompliance with the inclusion criteria (as to language, origin, availability or content) (Fig. 3; Supplementary Table 2). Based on phenotype details provided by the authors of five articles, three of them were included and two were excluded (Fig. 3). Hence, 84 articles of which fifteen reviews, three GeneReviews and one editorial, in addition to research articles and research letters, were finally included (Supplementary Table 3). Five selected articles describing studies that do not confirm the association between specific genes and the combination of OFCs and TA were also included and were classified as negative evidence.

From these 84 references, we identified 26 genes and 9 genomic loci presenting different types of evidences, ranging from borderline to significant associations even confirmed in animal models in some cases. The 26 candidate genes are described according to the evidence available in the current literature.

#### Msx1 and pax9

MSX1 and its main protein–protein interactor PAX9 are both transcription factors, members of the homeoprotein families which are co-expressed during craniofacial development and in different stages of tooth morphogenesis (Ogawa et al. 2005, 2006; Nakatomi et al. 2010). *MSX1* encodes a member of the muscle segment homeobox gene family, which acts as a transcriptional repressor during embryogenesis via the core transcription complex and other homeoproteins. MSX1 has been proven through mouse models and molecular and biochemical analyses on human tissues to play a main role in limb-pattern formation, tumor growth inhibition and craniofacial development, particularly in odontogenesis (EntrezGene; Davidson 1995; Lallemand et al. 2005; Park et al. 2005; Ogawa et al. 2006).

The MSX1 signaling loop also involves other essential homeobox genes, such as *BMP* genes, hence mediating the reciprocal epithelial–mesenchymal tissue interaction and regulating the development of both the craniofacial skeleton and the teeth (Zhang et al. 2002; De Coster et al. 2007). Although our systematic literature search did not identify any study proving evidence of association between *BMP* genes and the co-occurrence of the features discussed, it would be intriguing to further investigate this pathway, since the *BMP* gene family includes proposed OFC-causing genes (Ogawa et al. 2006; Lin et al. 2008; Suzuki et al. 2009; He et al. 2010; Suazo et al. 2010; Sahoo et al. 2011; Williams et al. 2012; Zawiślak et al. 2014; Liu et al. 2005) as well as genes involved in early tooth development, which disruption may result in tooth agenesis (Tompkins 2006; De Coster et al. 2007).

*MSX1* mutations are associated with the non-syndromic co-occurrence of CP and TA, especially HD, in humans (Table 1; Supplementary Table 4) (Carey and Viskochil 2002; Lidral and Reising 2002; Slayton et al. 2003; Vieira 2003; Wong and Hagg 2004; Modesto et al. 2006; Wilkie 2009; Kouskoura et al. 2011; Liang et al. 2012; Leslie and Marazita 2013). Similarly, *Msx1*-deficient mice exhibit severe craniofacial abnormalities, including clefting of the secondary palate and lack of teeth (Table 3) (Satokata and Maas 1994; Kavitha et al. 2010; Nakatomi et al. 2010).

Nowadays, a variable combination of selective TA with OFC (STHAG1) (OMIM# 106600) has been characterized in three affected members of a Dutch family whose genotyping revealed a heterozygous *MSX1* stop mutation inherited across generations (Table 1; Supplementary Table 4) (van den Boogaard et al. 2000). Later, a similar combined phenotype has been described as co-segregating with a different *MSX1* missense mutation in a Chinese family (Table 1; Supplementary Table 4) (Liang et al. 2012), supporting the hypothesis of the dual role of this gene in the etiology of TA and OFCs.

Even though *MSX1* mutations are known to cause non-syndromic OFCs and TA, Nieminen et al. (2003) described the case of a patient with Wolf–Hirschhorn syndrome (WHS) (OMIM# 194190) due to a complete deletion of the *MSX1* gene (Table 2; Supplementary Table 4), which is located in the deleted region in chromosome 4p, whose craniofacial features included CP as well as TA (Paradowska-Stolarz 2014).

**Table 2:** Genes contributing to the syndromic co-occurrence of OFCs and TA in presence of other phenotypes

Gene	Syndrome	Study	No. of cases	Type of OFC	Type of TA	TA location	Comments^a^	References
BCOR	Oculofaciocardiodental syndrome (OMIM# 300166)	Case series/literature review	2	CP/BF	HD/OD	Inside and outside	Two unrelated patients affected by different BCOR mutations	Feberwee et al. (2014)
FGFR1 (KAL2)	Kallman syndrome type 2 (OMIM# 147950)	Case series/literature review	2	CLP	OD	Inside and outside	Two patients reported in this study. Missing teeth: 52, 51, 61, 62, 72, 82 (pt. 2); 15,12,11,21, 47 45, 42, 32, 35 (pt. 6). In addition, a CLP-HD patient was found by through literature review (Pitteloud et al. 2006)	Bailleul-Forestier et al. (2010), Pitteloud et al. (2006)
	Kallman syndrome type 2 (OMIM# 147950)	Family-based study	1	CP	TA	Outside	One inherited (R622Q) and two de novo (C178S, R622G) mutations	Zenaty et al. (2006)
	Kallman syndrome type 2 (OMIM# 147950)	Family and unrelated case study	1	CP	TA	Outside	Proband II-2 shows CP and TA, along with an FGFR1 mutation	Xu et al. (2007)
	Kallman syndrome type 2 (OMIM# 147950)	Family-based study	1	CL/P	TA	Unclear	–	Tommiska et al. (2014)
	Kallman syndrome type 2 (OMIM# 147950)	Case–control study	1	CLP	TA	Unclear	–	Xu et al. (2015)
KISS1R	Hypogonadotropic hypogonadism with or without anosmia type 8 (OMIM# 614837)	Case–control study	1	CL	TA	Unclear	Gene proposed as a new candidate causative gene for Kallman syndrome	Xu et al. (2015)
IRF6	Van der Woude syndrome (OMIM# 119300)	Family-based study	3	CLP (1) CL (2)	HD	Inside and outside	–	Wienker et al. (1987)
	Van der Woude syndrome (OMIM# 119300)	Case report/series	2	CLP	HD	Unclear	The two affected subjects are brothers	Item et al. (2004)
	Van der Woude syndrome (OMIM# 119300)	Family-based study	22	CLP (14) CP (8)	HD	Unclear	Authors contacted to ask for further details. 12 VWS families showing OFCs and hypodontia in 22 members in total	Peyrard-Janvid et al. (2005, 2014)
	Van der Woude syndrome (OMIM# 119300)	Family-based study	3	CLP(2) CL(1)	HD	Unclear	The CL patient and the two CLP patients belong to two families with VWS	Ye et al. (2005)
	Van der Woude syndrome (OMIM# 119300)	Case report/series	1	CLP	HD	Outside	The affected subject of interest is the father of the proband. TA of 12 and 22	Minones-Suarez et al. (2012)
	Popliteal pterygium syndrome (OMIM# 119500)	Family-based study	1	CLP	HD	Unclear	Authors contacted to ask for further details. The affected subject belongs to a PPS family	Peyrard-Janvid et al. (2005, 2014)
KMT2D (MLL2)	Kabuki syndrome type 1 (OMIM# 147920)	Case report/series	1	CP	HD	Outside	–	David-Paloyo et al. (2014)
MSX1	Wolf–Hirschhorn Syndrome (OMIM# 194190)	Case report/series	1	CP	OD	Outside	Only patient exhibiting a deletion on MSX1 gene, with a ring-chromosome. TA of 18, 38, 48	Nieminen et al. (2003)
OFD1	Orofaciodigital syndrome type 1 (OMIM# 311200)	Family-based study	1	ARC	OD	Unclear	–	Shimojima et al. (2013)
PVRL1	Cleft lip/palate-Ectodermal dysplasia syndrome (OMIM# 225060)	Case report/series	1	CLP	HP	Unclear	–	Yoshida et al. (2015)
SATB2	Glass syndrome (OMIM# 612313)	Case report/series	1	CPO	OD	Outside	Small intragenic duplication affecting SATB2	Lieden et al. (2014)
	Glass syndrome (OMIM# 612313)	Case report/series	2	CP	OD	Unclear	1 Patient analyzed and re-interpretation of 1 case from the literature. Gene found disrupted because located in a translocation breakpoint	Rainger et al. (2014)
TBX1	Velocardiofacial syndrome (OMIM# 192430)	Case report/series	4	CP	TA	Outside	1 Patient has TA in the maxilla, 1 in the mandible and 2 in both jaws. Patient 1: 12, 22, 37. Patient 2: 15, 23, 25, 35, 41, 45. Patient 3: 15, 25. Patient 4: 32	Heliövaara et al. (2011)
TBX22	Cleft palate with ankyloglossia (OMIM# 303400)	Population-based study	1 (at least)	CLP	HD	Outside	Many patients have been reported and HD was also evaluated, but only for this patient the co-occurrence of CLP and HD (25 missing teeth) is clearly described	Kantaputra et al. (2011)
	Cleft palate with ankyloglossia (OMIM# 303400)	Case report/series	1	CLP	HD	Outside	The affected patient showed a TBX22 mutation causative of the phenotypes. 25 missing teeth	Kaewkhampa et al. (2012)
TP63	AEC syndrome	Case report/series	2	CLP	HD	Unclear	Unrelated patients affected by different TP63 mutations	Clements et al. (2010)
	AEC syndrome	Family-based study	1	CLP	HD	Unclear	–	McGrath et al. (2001)
TP63	Ectodermal dysplasia and B cell leukemia/lymphoma	Case report/series	1	CP	OD	Unclear	–	Cabanillas et al. (2011)
TP63	Ectrodactyly-ectodermal dysplasia-clefting (EEC) syndrome	Case report/series	4	CLP	HD	Unclear	Unrelated patients	Clements et al. (2010)
TP63	Ectrodactyly-ectodermal dysplasia-clefting (EEC) syndrome	Case report/series	2	OFCs	HD	Inside and outside	Unrelated patients	Yin et al. (2010)
TP63	ELA syndrome	Case report/series	1	CP	HD	Unclear	ADULT syndrome in combination with CPO (although usually not associated)	Prontera et al. (2011)
TP63	New (mixed spectrum: EEC, AEC and RHS)	Case report/series	1	CLP	HD	Unclear	HD of the deciduous dentition	Steele et al. (2005)
TWIST1	Proposed new syndrome	Case report/series	1	CP	HD	Unclear	Microdeletion affecting TWIST1 gene only	Busche et al. (2011)

*OFCs* orofacial clefts, *CL/P* cleft lip with or without cleft palate, *CL* cleft lip, *TA* tooth agenesis, *HD* hypodontia, *OD* oligodontia

^a^When available, the missing teeth are indicated in the comments column with the official enumeration

Mutations of PAX9, the main protein–protein interactor of MSX1, have also been described as potentially causative for combined OFCs and TA. Specifically, PAX9 is a member of the paired box family of transcription factors, which plays critical roles in embryogenesis, mainly skeletogenesis, tooth formation, palatogenesis and neural tube development (EntrezGene; Balling et al. 1996; Peters et al. 1998a; Hamachi et al. 2003; Hu et al. 2014; Monsoro-Burq 2015). Genetic disturbances of *MSX1* and *PAX9* are associated with TA, located both inside and outside the cleft area (Seo et al. 2013). In mouse, *Pax9* and *Msx1* are co-expressed during craniofacial development, and in double-mutant mice for these two genes, incompletely penetrant CL and absence of lower incisors have been reported (Table 3) (Nakatomi et al. 2010), suggesting that reduction of *PAX9* and *MSX1* gene dosage in humans may increase the risk for combined OFC and TA. However, this hypothesis was not confirmed in the study of Tallon-Walton et al. (2010).

**Table 3:** Genes contributing to OFCs and TA in mouse models

Gene	Mouse strain	Type of OFC	Type of TA	Comments	References
MSX1	Msx1^−/−^	CP	OD	Perinatal lethality in homozygous deficient mice	Satokata and Maas (1994)
	Msx1^−/−^	CP	TA	Also Msx1-Bmp4 transgene (Msx1^−/−^/Tg) mice were generated: the tooth agenesis was partially rescued and the palate appeared intact, although the rugae did not fuse at the midline	Zhang et al. (2002)
	Pax9^−/−^; Msx1^−/−^	CL	TA	The double-mutant mice show incompletely penetrant CL (38 % of cases) and lower incisors missing. Other genotypes were tested	Nakatomi et al. (2010)
PAX9	Pax9^flox/flox^;PGK-Cre Pax9^flox/flox^;Wnt1-Cre	CP	TA	Inactivation of Pax9 using Wnt1-Cre mice leads to CP (secondary palate) and TA and in other structures derived from neural crest cells	Kist et al. (2007)
	Pax9^−/−^;Msx1^−/−^	CL	TA	39 % of the mutants exhibit unilateral or bilateral CL while 100 % show the absence of teeth due to the lack of alveolar bones	Nakatomi et al. (2010)
PITX2	Pitx2^−/−^	CP	TA/OD	In human, this gene is causative of Axenfeld–Rieger syndrome type 1 (OMIM## 180500)	Lu et al. (1999), Kouskoura et al. (2011)
PTCH1	K14-Shh	OFCs	HD	In human, this gene is causative of Nevoid basal cell carcinoma syndrome (OMIM## 109400). Ptch1 encodes for the Shh pathway: the mice used as NBCCS model express Shh in basal epithelium under keratin-14 promoter	Cobourne et al. (2009)

*OFCs* orofacial clefts, *CL/P* cleft lip with or without cleft palate, *CL* cleft lip, *TA* tooth agenesis, *HD* hypodontia, *OD* oligodontia

Focusing on *PAX9* only, the first evidence of *PAX9* association with TA and OFCs arose from a *Pax9* ^−/−^ knockout mouse model described by Peters et al. (1998b), and was later confirmed in human by the study from Das et al. (2003) who reported a novel *PAX9* missense mutation and an exonic insertion in families with autosomal dominant TA where some of the members also showed CL/P (Table 3; Supplementary Table 4) (Kist et al. 2007; Kavitha et al. 2010).

#### Irf6

The *IRF6* gene encodes a member of the interferon regulatory transcription factor family; more specifically, the only member that is not related to immunological and inflammatory functions, but with morphogenesis, especially oral ectoderm and periderm formation, lip formation and spatio-temporal regulation of palatal shelf migration, adhesion and fusion (Richardson et al. 2009; Kousa and Schutte 2015). *IRF6* mutations are recognized as primary genetic causes of isolated and syndromic OFCs (Kondo et al. 2002; Zucchero et al. 2004; Blanton et al. 2005; Ingraham et al. 2006; Park et al. 2007; Beaty et al. 2010; Ludwig et al. 2012).

The most common OFC syndrome is the van der Woude syndrome (VWS) (OMIM# 119300), which represents 2 % of all syndromic CL/P. In 68 % of the cases, this syndrome is caused by *IRF6* mutations or deletions (Sander et al. 1995; Schutte et al. 1999; Kondo et al. 2002; de Lima et al. 2009). The dominant traits with variable expressivity and low penetrance are OFCs, HD and lip pits usually present in combination (Schinzel and Klausler 1986; Wienker et al. 1987). A number of studies describing *IRF6* missense, frameshift or stop mutations causing VWS in patients showing the co-occurrence of CL/P and/or CP and TA have been found in our literature search, resulting in a list of more than 33 cases, some of them belonging to VWS families (Table 2, Supplementary Table 4) (Vieira 2003; Wang et al. 2003; Ghassibé et al. 2004; Item et al. 2004; Wong and Hagg 2004; Ye et al. 2005; Peyrard-Janvid et al. 2005; Minones-Suarez et al. 2012; Klein et al. 2013; Peyrard-Janvid et al. 2014). As further confirmation, another case report presented two patients with VWS belonging to the same family with the typical features of this syndrome, including both CL/P and HD. However, in this specific case, the gene appears fully missing as encompassed by a large deletion inherited in the affected members of this family (Wong et al. 1999) (Table 4; Supplementary Table 6). Since this deletion, del(1)(q32), encompasses 198 genes in total, the contribution of other genes located within the deleted region cannot be excluded (Supplementary Table 6).

**Table 4:** Genomic loci associated with OFCs and TA in human

Gene	Study	No. of patients	Type of OFC	Type of TA	TA location	Comments	References
1q21–q25	Case report/series	1	CLP	OD	Unclear	The reported patient exhibits a del(1)(q21–q25)	Schinzel and Schmid (1980)
1q32	Family-based study	2	CL/P	HD	Inside and outside	The patients are affected by Van der Woude syndrome (OMIM# 119300), with del(1)(q32)	Wong et al. (1999)
2q31.2–q33.2	Case report/series	1	CP	OD	Outside	Analysis of CNVs by CGH showed in this patient a del(2)(q31.2–q33.2). Proposed new syndrome	Rifai et al. (2010)
4p16.3	Case report/series	1	CP	OD	Unclear	The patient is affected by Wolf–Hirschhorn syndrome (OMIM# 194190)	Maas et al. (2008)
8q24	Case–control/Family-based study	31	OFCs	TA	Outside	The locus contains an SNP (rs987525) significantly associated with OFCs and TA	Yildirim et al. (2012)
16q22	Case report/series	4	CP	OD	Inside and outside	All the patients belong to the same family. Three of them present a fragile site in 16q22	Bettex et al. (1998)
	Case report/series	1	CP	HD	Outside	The patient, affected by oropalatal Bettex–Graf dysplasia, showed a fragile site in 16q22	Janiszewska-Olszowska et al. (2013)
	Case report/series	1	CP	OD	Outside	The patient shows a fragile site in 16q22 and features similar to those of Bettex–Graf dysplasia	McKenzie et al. (2002)

*OFCs* orofacial clefts, *CL/P* cleft lip with or without cleft palate, *CL* cleft lip, *TA* tooth agenesis, *HD* hypodontia, *OD* oligodontia

In contrast, a study by Ali et al. (2009) failed to report the association between *IRF6* markers and this syndrome in a cohort of Indian VWS families, supporting the evidence that other genes may contribute to the etiology of this syndrome, such as *GHRL3*.

Apart from the VWS, different mutations in the same gene lead to another syndrome associated with OFCs, named popliteal pterygium syndrome (PPS) (OMIM# 119500) (Kondo et al. 2002), which shares some clinical features of VWS with the addition of webbed skin of the legs, genital malformations and oral synechiae. From our literature search, a PPS family was found based on the combination of OFC and TA in one affected member due to an inherited *IRF6* mutation (Table 2; Supplementary Table 4) (Peyrard-Janvid et al. 2005, 2014).

Furthermore, the contribution of *IRF6* variation to non-syndromic OFCs has been sturdily proven. Originally, a GWAS study identified the *IRF6* region as a susceptibility locus for non-syndromic OFCs (Beaty et al. 2010), which has been later confirmed by several further studies in human and in mouse models. The role of *IRF6* in non-syndromic OFCs in combination with TA located outside the cleft area was thoroughly investigated by Letra et al. (2012) in a cohort of 134 Brazilian patients affected by both these conditions, thus identifying a borderline-associated *IRF6* marker (rs658860) in the sub-group of subjects showing CP and TA (Table 1; Supplementary Table 4). As further evidence, a statistically significant association was found between co-occurring OFCs and TA and an SNP in the AP-2α binding site of the *IRF6* promoter in a large study based on 93 Latvian patients with isolated OFCs (Table 1) (Krasone et al. 2014). On the contrary, Pegelow et al. (2008) did not find any significant association between different *IRF6* SNPs and non-syndromic CL/P in 17 Swedish OFC families that included 13 members affected with OFC, further supporting the hypothesis of a minor contribution of other genes to the pathogenesis of these conditions.

#### Tp63

The *IRF6* gene is one of the main targets of another transcription factor, p63 (tumor protein 63). Disruption of a p63-binding site upstream to *IRF6* due to a small insertion has been seen to cause VWS in a family where the *IRF6* gene was not mutated (Fakhouri et al. 2014), proving that the syndrome may be caused by an upstream disruption which does not directly affect the causative gene sequence.

*TP63* encodes for a member of the p53 family of transcription factors, named p63, for which unlike p53, a role in tumorigenesis has not been defined so far, while its role in proliferation, development and commitment to stratified epithelial tissues has been extensively characterized in humans as well as in animal models (EntrezGene; UniProt; Yang et al. 1998). *Tp63* ^−*/*−^ knockout mice show typical developmental defects in epithelium-related structures including skin, hair, limbs, palate and mammary glands (Mills et al. 1999; Yang et al. 1999). In humans, the disruption of *TP63* regulation leads to abnormalities of the skin, the limb and the orofacial structure, resulting from the impaired transcription of its targets which include not only *IRF6* but also other cleft-associated genes, such as *TFAP2α* and *RIPK4* (McDade et al. 2012; Mitchell et al. 2012). Mutations in the *TP63* gene itself have been associated with multiple syndromes, called p63 syndromes: ectrodactyly-ectodermal dysplasia-clefting (EEC) (OMIM# 129900), split-hand/foot malformation type 4 (SHFM4) (OMIM# 605289), ankyloblepharon-ectodermal dysplasia-cleft syndrome (AEC) (OMIM# 106260), acro-dermato-ungual-lacrimal-tooth syndrome (ADULT) (OMIM# 103285), limb-mammary syndrome (LMS) (OMIM# 603543) and Rapp–Hodgkin syndrome (RHS) (OMIM# 129400). Of these, the EEC syndrome most frequently shows co-occurrence of OFCs and TA (Itin and Fistarol 2004; Kouskoura et al. 2011; Tadini et al. 2013). In our systematic search, *TP63* mutations have been seen to likely contribute to the syndromic co-occurrence of TA and OFCs, in relation to different p63 syndromes.

In 2010, two studies described novel *TP63* mutations in six patients with EEC exhibiting OFCs and HD (Table 2; Supplementary Table 4) (Clements et al. 2010; Yin et al. 2010). One year later, an editorial by Sripathomsawat et al. (2011) reviewed two Thai patients with EEC and six previously published Dutch families focusing mainly on the oral and dental features, with particular attention on OFCs and TA.

Cabanillas et al. (2011) characterized one patient showing a combination of B cell leukemia and ectodermal dysplasia including CP and TA, theoretically caused by a pathogenic maternally inherited heterozygous germline mutation of the *TP63* gene (Table 2; Supplementary Table 4). The review by Tadini et al. (2013) focused on *TP63*-related diseases, describing CL/P and TA or anodontia (AD) as a typical feature of RHS while CP with or without bifid uvula and TA as a hallmark of LMS syndrome. The core clinical features of the LMS were defined upon the investigation of a large Dutch family, in which affected individuals were characterized by severe limb and gland anomalies, CP and TA (van Bokhoven et al. 1999). The genetic defect was mapped to the subtelomeric region of chromosome 3q, which led to the identification of causative *TP63* mutations in EEC syndrome, and subsequently related conditions including LMS.

Another syndrome-causing *TP63* mutation was defined by McGrath et al. (2001) who reported on an AEC family with phenotypes including CLP and TA due to a *TP63* missense mutation, later confirmed in a case report by Clements et al. (2012) describing an AEC patient with a CLP and TA (Table 2; Supplementary Table 4). Intriguingly, Clements et al. (2010) proposed that RHS and AEC represent a variable spectrum of the same genetic disorder, investigating four cases of which two showed bilateral CLP and TA due to two missense mutations of *TP63* gene (Table 2; Supplementary Table 4). Interestingly, a case report described a patient with ADULT syndrome-like phenotype associated with CP and TA, who was found to be heterozygous for a *de novo* mutation in *TP63* (Table 2; Supplementary Table 4) (Prontera et al. 2011). The peculiar aspect of this case is represented by the unusual combination of features: ADULT differs from EEC and LMS mainly by the absence of CL/P, but in this case CP was also present, thus the authors suggested to combine the three phenotypic spectra into a unique syndrome called ELA (Prontera et al. 2011). Patients with mixed phenotypic variations seen in EEC, AEC and RHS were previously described by Steele et al. (2005), one of these showed CLP and TA in addition to other anomalies, resulting from another *TP63* SNP (Table 2; Supplementary Table 4) (Steele et al. 2005). The new and variable phenotypic features noted in these patients emphasize the wide spectrum of diseases caused by mutations in *TP63*.

#### The TGF pathway

The transforming growth factors (TGFs) represent a large family of proteins whose members regulate a remarkable range of biologic processes by acting on the transcription of genes controlling cell proliferation, differentiation, death, adhesion, migration and positioning. This superfamily is further divided into two classes, TGFα and TGFβ, which are not structurally nor genetically related but both modulating similar cell responses through different receptor mechanisms (TGF preferentially with EGF receptor, EGFR, while TGFβ via TGFβ receptors, TGFβRs) (Brachmann et al. 1989; Wong et al. 1989; Wrana et al. 1994; Heldin et al. 2009; Macias et al. 2015).

One of the most well-characterized members of the TGFβ subfamily is TGFβ3, a secreted protein that plays an essential role in embryogenesis by modulating mesenchymal cell proliferation, differentiation, migration and extracellular matrix production, via transmembrane TGFβRs which then transduce the signal from the cell surface to the cytoplasm mainly via SMAD proteins (EntrezGene; Wrana et al. 1994; Derynck and Zhang 2003; Massagué et al. 2005; Derynck et al. 2014; Macias et al. 2015). Diseases associated with *TGFβ3* mutations include Loeys–Dietz syndrome-5 (LDS5) (a.k.a. Rienhoff syndrome) (OMIM# 615582) and arrhythmogenic right ventricular dysplasia (OMIM# 107970).

In the literature, OFCs with TA outside the cleft region was found to be positively associated with *TGFβ3* variants, compared with non-OFC controls (Slayton et al. 2003). This evidence has been confirmed also in animal models, where mutant mice for *TGFβ3* have been described as affected by HD and CP (Table 3) (Vieira 2003).

*TGFβ3* represents one of the main ligands of two serine/threonine protein kinase receptors, *TGFβR1* and *TGFβR2*, which have also been investigated in relation to syndromic OFCs (Loeys et al. 2005). Moreover, these genes have been associated with Marfan syndrome (OMIM# 154700), Loeys–Dietz syndrome (LDS) (OMIM# 609192; OMIM# 610168), features of which include CP (Loeys et al. 2005), and Kallmann syndrome (KAL, a.k.a. hypogonadotropic hypogonadism with anosmia) (OMIM# 147950). Interestingly, a study based on 14 patients with KAL whose phenotypic spectrum includes CP and tooth anomalies, found causative non-exonic mutations in *TGFβR1* and *TGFβR2* (Table 2; Supplementary Table 4). Although it is not specified whether TA is included in the analyzed dental abnormalities, this evidence remains interesting since patients with KAL share phenotypes with patients suffering from LDS type 2, suggesting a possible minor role for the TGFβR-mediated pathway in KAL (Bottani et al. 2006).

Unlike *TGFβ3*, *TGFα* encodes a ligand for EGFR that works synergistically with the TGFβ pathway to regulate cell proliferation, differentiation and embryonic development (Brachmann et al. 1989; Wong et al. 1989). A variety of positive and negative results have been reported concerning the association between OFC and *TGFα*, which is highly expressed in the medial edge epithelium of the palatal shelves at the time of palatal fusion (EntrezGene; Letra et al. 2012). Variants in *TGFα* have also been described as a possible risk factor for OFCs in case of maternal exposure to cigarette smoke, alcohol consumption or improper retinoic acid intake (Ardinger et al. 1989; Chenevix-Trench et al. 1992; Feng et al. 1994; Shaw et al. 1996a, b; Pezzetti et al. 1998; Jugessur et al. 2003; Zeiger et al. 2005; Letra et al. 2012). In addition, previous evidences have suggested that a possible interaction between *IRF6* and *TGFα* may contribute to TA (Vieira et al. 2007).

In our literature search, a case–control study based on the genotyping of 406 Brazilian Caucasian patients with non-syndromic OFC (106 affected by TA) found a significant association between *IRF6* as well as *TGFα* markers and the combination of OFCs and TA (Table 1; Supplementary Table 4) (Letra et al. 2012), representing a further clue of a possible role of *TGFα* in the dual pathogenesis of these orofacial defects.

#### Satb2

Originally identified as *KIAA1034*, *SATB2* encodes a transcription regulator and chromatin remodeling factor, belonging to the homeobox proteins (*SATB* Homeobox 2). Its expression starts in the embryo and is later conserved in adult tissues, such as the spinal cord, the kidneys, and the central nervous system (UniProtKB; Zhao et al. 2014). This homeobox protein acts in concert with the BMP signaling pathway to modulate skeletogenesis by triggering several critical transcription factors like *RUNX2*, the master and the earliest osteogenic transcription factor (Zhao et al. 2014). A number of studies confirmed that *SATB2* is strongly expressed in the developing craniofacial regions during mammalian embryogenesis, where it regulates osteoblast differentiation and craniofacial patterning determination (Britanova et al. 2006; Dobreva et al. 2006; Zhao et al. 2014). Consequently, mutations of this gene lead to increased apoptosis in the craniofacial mesenchyme and to impaired expression patterns of three genes, *PAX9*, *ALX4* and *MSX1*, implicated in the regulation of craniofacial development in humans and mice, resulting in facial clefts (Dobreva et al. 2006; Zhao et al. 2014). In a large number of studies, the contribution of *SATB2* variants to OFCs in human has been confirmed, especially CP, both in non-syndromic OFC (OMIM# 119530) as well as in syndromes such as Glass syndrome (OMIM# 612313), and Pierre Robin sequence with or without ankyloglossia and cleft-associated intellectual disability (OMIM# 261800) (FitzPatrick et al. 2003; Beaty et al. 2006; Britanova et al. 2006; Leoyklang et al. 2007; Rosenfeld et al. 2009; Urquhart et al. 2009; Rainger et al. 2014). In addition, recent evidence suggests a possible link between *SATB2* and dental anomalies including TA (Rosenfeld et al. 2009; Kaiser et al. 2015). Regarding the co-occurrence of these pathogenic conditions, a case report describes a male patient with multiple associated phenotypes, including CP and TA, who carries a small intragenic duplication in the *SATB2* gene affecting three coding exons (Table 2; Supplementary Table 4) (Lieden et al. 2014). In addition, the heterozygous loss-of-function mutations of *SATB2* have been seen to result in micrognathia and CP both in mice and humans. In a recent study, two patients both affected by CP and TA were described with translocations, the breakpoints in which were mapped to *SATB2* and *PLCL1*, t(2;11)(q33.1;p13) and t(1;2)(p34;q33), further supporting the hypothesis of a causative role of *SATB2* in a common etiologic mechanism shared between OFCs and TA (Table 1; Supplementary Table 4) (Rainger et al. 2014).

#### Tbx22

A highly conserved gene family involved in the embryonic patterning from *Drosophila* to vertebrates is the T-box family, whose members are derived from events of gene duplication and cluster dispersion (Packham and Brook 2003).The key role played by TBX proteins during many aspects of embryonic development has been demonstrated by the generation of targeted T-box gene deletions in zebrafish and mouse (Bollag et al. 1994; Agulnik et al. 1996; Packham and Brook 2003). These models confirm that TBX factors are responsible for the decision of paraxial mesoderm to follow a mesodermal or neuronal pathway (Chapman and Papaioannou 1998). Due to its essential role in human palatogenesis, mutations in one of the TGF members, *TBX22*, have been reported in patients with OFCs and TA as well as in OFC-associated syndromes, such as inherited X-linked cleft palate with ankyloglossia (OMIM# 303400) and in Abruzzo–Erickson syndrome (OMIM# 302905) (Braybrook et al. 2001, 2002; Herr et al. 2003; Marçano et al. 2004; Suphapeetiporn et al. 2007; Kim et al. 2009; Pauws et al. 2009, 2013; Acevedo et al. 2010; Kantaputra et al. 2011; Kaewkhampa et al. 2012; Gurramkonda et al. 2015). The speculation about its contribution to OFCs and TA originated from two sources. First, one individual was found to present both OFC and TA likely due to a *TBX22* missense mutation in a study based on a large cohort of patients with ankyloglossia and patients with sporadic isolated OFC (Table 2; Supplementary Table 4) (Kantaputra et al. 2011). Second, a case report describing a male patient with complete unilateral CLP and TA identified a hemizygous missense mutation in *TBX22* (Table 1; Supplementary Table 4) (Kaewkhampa et al. 2012).

#### Chd7 and fgfr1/fgf8

On chromosome 8, two specific loci, 8p11.23 and 8q12.2, have been associated with the etiology of Kallmann syndrome (a.k.a. hypogonadotropic hypogonadism type 2 with anosmia) (OMIM# 147950) whose minor phenotypic manifestations include OFC and TA (Layman 2013). These two loci encompass two genes, proposed as causative genes of KAL: *FGFR1* and *CHD7*, respectively (Beate et al. 2012; Layman 2013).

Located in 8q12.2, *CHD7* gene encodes a DNA-binding protein that acts as a positive transcriptional regulator by binding to enhancer elements in the nucleoplasm, and its disruption leading to Kallmann syndrome or CHARGE syndrome (OMIM# 214800).

The other locus, 8p11.23, contains other genes, including *FGFR1* (a.k.a. *KAL2*) and *FGF8*, both considered as main players in Kallmann syndrome. Mutations in *FGFR1* are also described as causative for other syndromes, some of them including OFCs and dental anomalies (Kim et al. 2005; Riley et al. 2007; Stoler et al. 2009; Simonis et al. 2013), like a gain-of-function *FGFR1* mutation associated with Kallmann syndrome and loss-of-function mutations in craniosynostosis presenting OFCs (Dodé et al. 2003). FGFR1 is a member of the fibroblast growth factor receptor (FGFR) family, a group of tyrosine kinase receptors belonging to the FGF pathway, which regulates a wide range of cell responses, such as angiogenesis, cell migration, and embryonic development, including skeletal formation (EntrezGene; Muenke and Schell 1995). This FGF signaling pathway contains also the ligands of these receptors, such as FGF8. Interestingly, *FGFR1* as well as *FGFR2* are well-characterized OFC-associated genes, but have been only recently investigated for possible involvement in TA (Huang et al. 2015; Hosokawa et al. 2009).

Rare sequence variants (defined as genetic variants with a minor allele frequency lower than 1 % in control populations) in *FGFR1* (10 %) and *CHD7* (6 %) are the most common autosomal causes of Kallmann syndrome, whereas another causative gene, *KAL1*, has been estimated to have a prevalence of 5–10 % in affected males (X-linked recessive) (Layman 2013). Costa-Barbosa et al. (2013) performed a detailed phenotypic comparison in a large group of 151 KAL subjects harboring known rare sequence variants, in eight genes belonging to six molecular pathways, which included *CHD7* and *FGFR1/FGF8*. The co-occurrence of TA and OFC was observed in only two patients with rare sequence variants affecting *CHD7* (Table 2; Supplementary Table 4), and although interesting as a clue suggesting the existence of a connection between the gene and the phenotypes of interest, the low number of cases was not sufficient to emerge as a statistically significant phenotype predictor. In contrast, among patients with CL/P, 54 in total, a significant association resulted in the sub-group of patients with CL/P showing TA (39 %) and mutations in the *FGF8/FGFR1* (Table 2; Supplementary Table 4). Albuisson et al. (2005) studied a cohort of 98 patients with Kallmann syndrome, seven of whom contained mutations in *FGFR1* related to OFCs and TA: of these, no one has been reported with the combined phenotypes; however, two patients with different FGFR1 mutations (p.D129A and p.V273 M) showed CP while another patient (c.1093_1094delAG) showed TA. Although no patients showed the combination of the phenotypes in this cohort, the study still raises interesting hypothesis since the same gene is affected and apparently related to both TA and OFCs even if in different subjects. Altogether, in our search we identified seven *FGFR1* mutations that have been proposed as causative in seven patients with Kallmann syndrome, exhibiting CL/P and TA among other main phenotypes (Supplementary Table 4) (Zenaty et al. 2006; Xu et al. 2007, 2015; Bailleul-Forestier et al. 2010; Tommiska et al. 2014), representing relevant insights into a possible common *FGFR1*-related mechanism that may contribute to the dual etiology of OFCs and TA.

#### The WNT signaling pathway

The wingless-type MMTV integration site family (Wnt family) consists of structurally related genes encoding secreted signaling proteins implicated in several developmental processes, such as cell fate regulation and patterning during embryogenesis (EntrezGene; Dale 1998; Yin and Bian 2015). Together with the TGFβ signaling pathway, the canonical Wnt/β-catenin pathway provides most genes related to the network active during the initiation phase of palatogenesis and odontogenesis (Smalley and Dale 1999; Bae et al. 2015; Yin and Bian 2015). At the same time, Wnt signaling has been confirmed as implicated in oncogenesis at a later stage of life by a large number of studies since the late 1990s (e.g., Dale 1998; Morin 1999; Smalley and Dale 1999). In the last decade, mutations affecting the WNT10A member of this family have emerged as frequent causes of syndromic as well as non-syndromic TA (van den Boogaard et al. 2012; Arte et al. 2013; He et al. 2013; Abdalla et al. 2014; Alves-Ferreira et al. 2014; Kantaputra et al. 2014; Mues et al. 2014; Song et al. 2014; Vink et al. 2014). In addition, a *WNT10A* polymorphism is described to be associated with a significantly increased risk for OFC in a Chinese cohort (Feng et al. 2014; Beaty et al. 2006). However, unlike *WNT3* and *WNT5*, no studies currently published have investigated *WNT10A* gene in relation to these combined orofacial phenotypes.

Mutations in *WNT3* are well-known causes of syndromic tetra-amelia with CLP (OMIM# 273395), but the disruption of this gene has recently also been described as involved in non-syndromic OFC with TA (Table 2; Supplementary Table 4) (Yao et al. 2011; Mostowska et al. 2012). Interestingly, Menezes et al. (2010) identified a significant association between a marker located close to *WNT3* gene in the group of patients affected by bilateral CL/P and agenesis of the lateral incisors. Specifically, this point mutation (rs142167, personal communication) is located in the intronic sequence of *NSF*, a gene flanking *WNT3* and encoding a transporter involved in the vesicle-mediated trafficking within the Golgi cisternae (UniProt). However, since the effect of this mutation via *NSF* or through the close *WNT3* gene is still not clear, further investigations are needed. In the same gene family, *WNT5A* has been reported by Person et al. (2010) as the causative gene of autosomal Robinow syndrome (ADRS) (OMIM# 180700) and in a recent update Roifman et al. (2015) described TA as a typical feature and CLP as a less common phenotype. Furthermore, mutations in other canonical WNT signaling-related genes have been shown to cause either TA with or without OFCs or other associated disorders, such as *AXIN2*, playing an important role in the regulation of β-catenin stability in the cytoplasm, and *LRP6* functioning as a transmembrane co-receptor of Frizzled proteins (EntrezGene; Sarkar and Sharpe 1999; Bodine and Komm 2006). For *LRP6*, its role in lip formation and odontogenesis has been studied in mice and in patients (Song et al. 2009; Massink et al. 2015; Ockeloen et al. 2016) while the role of *AXIN2* is not yet fully defined although its involvement in embryogenesis and oncogenesis is clear. Intriguingly, a pathogenic *AXIN2* mutation has been described as causative for both TA and cancer development in a Finnish family where the TA phenotype segregated with colorectal cancer predisposition (Lammi et al. 2004). In a case–control study including 500 patients with non-syndromic OFC and 500 unrelated controls, an *AXIN2* polymorphism (Table 1, Supplementary Table 4) showed association (*rs7591*, *p* = 0.01) with the co-occurrence of unilateral right CL/P with TA, stimulating the interest in this gene that may be involved in both pathogenic processes (Letra et al. 2009).

#### Cdh1

*CDH1* (cadherin 1) belongs to the cadherin superfamily of transmembrane adhesion proteins, which play important roles in craniofacial morphogenesis (Taneyhill 2008), specifically during the formation of facial cartilages and bones as well as during dental development (Verstraeten et al. 2010), either by controlling cell–cell adhesion or interacting with Wnt intracellular signaling (Di Benedetto et al. 2015; Schambony et al. 2004; Bienz 2005; Brembeck et al. 2006). To date, mutations affecting this gene have been described in families presenting a combination of gastric cancer and CL/P (Letra et al. 2009; Frebourg et al. 2006; Vogelaar et al. 2013). In a wide case–control study, 500 Brazilian patients with OFC and 500 unrelated controls were analyzed to investigate the role of *CDH1* and *AXIN2* markers in OFC etiology. Interestingly, the sub-group of patients with OFC showing also TA, considered as cleft sub-phenotype in this study, revealed an association of one *CDH1* marker (rs11642413, *p* = 0.008) and one AXIN2 marker (*rs7591*, *p* = 0.01) with unilateral right CL/P (Table 1; Supplementary Table 4) (Letra et al. 2009).

### Other candidate genes rarely associated with co-occurrence of orofacial clefting and tooth agenesis

#### Kmt2d and kdm6a

*KMT2D* (a.k.a. *MLL2*), which encodes an SET-domain-containing protein of lysine-specific histone methyltransferases responsible for trimethylation of histone H3 at lysine 4 (H3K4me3), and *KDM6A*, a histone H3 lysine 27 (H3K27)-specific demethylase, have been recognized as the main causative genes of Kabuki syndrome (a.k.a. Niikawa–Kuroki syndrome) (OMIM# 147920, OMIM# 300867, respectively). These two enzymes modulate the gene expression by epigenetic modifications, playing a critical role in craniofacial, heart and brain development (Van Laarhoven et al. 2015). *KMT2D*-related Kabuki syndrome (type 1) (OMIM# 147920) is inherited in an autosomal dominant manner and *KMT2D* mutations are present in 34–76 % of patients with KS, while *KDM6A*-related KS (type 2) (OMIM# 300867) is less frequent and inherited in an X-linked manner (Adam et al. 1993; Van Laarhoven et al. 2015). This syndrome has peculiar craniofacial phenotypes, including as minor CL/P features, hypodontia and lower lip pits in some cases, which can lead to a misdiagnosis of VWS (Matsumoto and Niikawa 2003; David-Paloyo et al. 2014).

Patients with a *KMT2D* mutation are more likely to have the distinctive Kabuki facial phenotype, which may reflect the fact that a portion of those without a *KMT2D* mutation may have been misdiagnosed. However, in the literature, molecular analyses confirmed the presence of a *KMT2D* mutation in only one patient with KS exhibiting the co-occurrence of CP and HD (Table 2; Supplementary Table 4) (David-Paloyo et al. 2014).

#### Ofd1

The X-linked gene *OFD1* has been recognized as a causative gene of the oral–facial–digital syndrome type 1 (OFD1) (OMIM# 311200) (Klein et al. 2013). This gene encodes a centrosomal protein implicated in embryonic development by regulating the canonical Wnt signaling pathways and the sonic hedgehog (Shh) signal during the early embryonic specification of the left–right axis in mammals (EntrezGene; Macca and Franco 2009). In OFD1 syndrome, CP is present in more than 50 % of the affected patients, and also another minor OFC subtype, the cleft alveolus, is commonly reported in patients with OFD. In addition, the lower lateral incisors are missing in 50 % of the individuals, which is also associated with fibrous bands in the region (Klein et al. 2013). In their NCBI GeneReview, Toriello and Franco (1993) indicate that in OFD1 mainly median clefts or (pseudo)clefts of the upper lip are present. In a case series found in our search, two *OFD1* siblings sharing the same mutation were described; only one of them had TA and a cleft alveolar ridge (Table 2; Supplementary Table 4) (Shimojima et al. 2013).

#### Bcor

The *BCL6* corepressor gene, *BCOR*, encodes a protein that inhibits gene expression by sequence-specific DNA-binding proteins such as BCL6 and MLLT3 when recruited to their promoter regions (UniProt). In addition, this gene is known to interact with *AP*-*2α*, a known OFC gene (Milunsky et al. 2008; Rahimov et al. 2008), and *WNT10A*, a known TA gene. Syndromes associated with variants in *BCOR* include oculofaciocardiodental syndrome (OFCD) (a.k.a. syndromic microphthalmia type 2, OMIM# 300166), which is known to be associated with both OFC and TA. In a review by Kantaputra (2014), the OFCD syndrome has been described to be associated with several dental and orofacial anomalies including HD and craniofacial features including CP. As confirmation, Feberwee et al. (2014) indeed found two patients affected with OFCD carrying *BCOR* point mutations, one affected by CP and mild HD while the other by CP and OD, although a concrete evidence that these two phenotypes are ‘typical’ features of OFCD syndrome is lacking (Table 2; Supplementary Table 4).

#### Twist1

Within the basic helix–loop–helix (bHLH) transcription factor family, which plays an essential role in cell lineage determination and differentiation, *TWIST1* (twist family BHLH transcription factor 1) was found by Busche et al. (2011) as the only gene affected by a microdeletion of 7p21 in three patients (Table 2; Supplementary Table 4). Although a wide range of phenotypes was present in these subjects, such as features resembling typical traits of blepharophimosis–ptosis–epicanthus inversus syndrome (BPES) (OMIM# 110100) and Saethre–Chotzen syndrome (OMIM# 101400), CP and TA were present in one of these patients.

#### Pitx2

A transcriptional regulator, member of the PITX homeobox family, is encoded by *PITX2* (paired-like homeodomain 2) and is involved in the morphogenesis of the eyes, the teeth and abdominal organs (EntrezGene). Mutations in this gene are associated with Axenfeld–Rieger syndrome type 1 (RIEG1) (OMIM# 180500), iridogoniodysgenesis syndrome type 2 (IRID2) (OMIM# 137600), and sporadic cases of Peters anomaly (OMIM# 604229).

Main characteristics of Axenfeld-Rieger syndrome type 1 (RIEG1) include severe TA that is associated with midfacial hypoplasia and CP (Kavitha et al. 2010). Although evidences in humans currently lack, *Pitx2* knockout mice typically exhibit TA, CP and abnormal development of the maxilla and mandible (Table 3) (Kouskoura et al. 2011), supporting the hypothesis of a conserved relation between this gene and orofacial defects in human.

#### Ptch1

In our literature search, patched 1 (*PTCH1*) gene encoding a member of the patched family which functions as a receptor for Indian hedgehog (IHH), desert hedgehog (DHH) and mainly for sonic hedgehog (SHH) was also identified. Shh represents a key inductive signal for a variety of patterning events that take place in the early embryo, and consequently PTCH1 is also involved in embryonic development. Interestingly, mutations in *SHH* cause holoprosencephaly (OMIM# 142945), whose wide phenotypic spectrum also includes CL/P and the presence of a single median upper central incisor, which may be considered as a mild form of TA (Roessler et al. 1996; Orioli et al. 2002).

A study based on a transgenic mouse model expressing Shh, ligand of *Ptch1*, in basal epithelium under the control of a specific Keratin-14 promoter showed that an increased activity of Shh in this tissue prevents apoptosis, palatal shelf fusion and tooth development at the bud stage (Table 3) (Cobourne et al. 2009). *PTCH1* is one of the causative genes for nevoid basal cell carcinoma syndrome (a.k.a. basal cell nevus syndrome, OMIM# 109400), which includes OFC and TA as secondary features of its core characteristics, including also jaw cysts, basal cell tumors and skeletal abnormalities (Cobourne et al. 2009; Lam et al. 2013).

#### Pvrl1

Mutations in *PVRL1*, encoding an adhesion protein contributing to the adherent and tight junction formation in epithelial and endothelial cells, are known to cause CL/P-ectodermal dysplasia syndrome (CLPED, a.k.a. Zlotogora syndrome) (OMIM# 225060) as well as non-syndromic CL/P (EntrezGene; Suzuki et al. 2000; Sözen et al. 2001; Turhani et al. 2005; Avila et al. 2006; Scapoli et al. 2006; Sözen et al. 2009). So far, combined CL/P and HD has only been diagnosed in one CLPED patient who exhibited a homozygous nonsense mutation in the *PVRL1* gene (Supplementary Table 4) (Yoshida et al. 2015).

#### Kiss1r

*KISS1R* gene encodes for a galanin-like G protein-coupled receptor that plays a role in endocrine function regulation and puberty onset by binding its ligand, metastin, and triggering a signaling via phospholipase C and G(q) proteins (EntrezGene; UniProtKB). This gene is known as the causative gene of hypogonadotropic hypogonadism type 8 with or without anosmia (OMIM# 614837) (Acierno et al. 2003; de Roux et al. 2003; Brioude et al. 2013). Interestingly, mutations of *KISS1R* have recently been linked to Kallmann syndrome: specifically, Xu et al. (2015) reported on a Kallmann patient exhibiting CL and TA (Table 2; Supplementary Table 4). Although a single evidence is not enough to draw any conclusion, the relation of the *KISS1R* mutation with Kallmann syndrome including the co-occurrence of OFC and TA is worth to be further investigated.

### GO term analysis and gene clustering

To find the hypothesized common etiological genetic factors explaining the co-occurrence of TA and OFC, we further analyzed the data as follows using a Gene Ontology (GO) term mapping tool. The GO terms related to the biological processes mediated by the 26 candidate genes were mapped to 51 broad categories, which were subsequently combined to generate six super-clusters (Supplementary Table 5): (a) anatomical development, (b) cell division, growth and motility, (c) cell metabolism and catabolism, (d) cell transport, (e) cell structure organizations and (f) organ/system-specific processes.

Anatomical development, the first cluster, includes a total of 23 genes related with embryogenesis, morphogenesis, anatomical structure formation and growth (in alphabetical order): *AXIN2, BCOR, CDH1, CHD7, FGF8, FGFR1, IRF6, KDM6A, KMT2D, MSX1, OFD1, PAX9, PITX2, PTCH1, PVRL1, SATB2, TGFβ3, TGFβR1, TGFβR2, TP63, TWIST1, WNT3,* and *WNT5A*. Cell division, growth and motility, the second cluster, largely overlaps with the first cluster, encompassing 23 genes involved in different processes that range from cell division and proliferation, over differentiation, to cell motility and adhesion. Excluding *PAX9* and *BCOR*, the other 21 genes of the first cluster are present also in the second, which in addition includes *TGFα* and *KISS1R*. Similarly, 23 genes are included in the third cluster, for cell metabolism and catabolism: *AXIN2, BCOR, CDH1, CHD7, FGF8, FGFR1, IRF6, KDM6A, KISS1R, KMT2D, MSX1, PAX9, PITX2, PTCH1, SATB2, TBX22, TGFα, TGFβ3, TGFβR1, TGFβR2, TP63, TWIST1,* and *WNT5A*. Other biological processes highly represented in our set of candidate genes are the cell transport and signal transduction, comprising 21 genes which correspond to the second cluster excluding *SATB2* and *OFD1*. In addition, 17 candidate genes are also implicated in the cellular structure organization, specifically membrane formation and cytoskeleton assembly: *AXIN2, BCOR, CDH1, CHD7, KMT2D, KDM6A, OFD1, PTCH1, PVRL1, SATB2, TGFα, TGFβ3, TGFβR1, TP63, TWIST1,* and *WNT5A*. The last cluster includes 17 genes contributing to organ/system-specific processes, such as the immune system, the neurological system and the circulatory system processes: *CDH1, CHD7, FGF8, FGFR1, IRF6, KMT2D, MSX1, PITX2, PVRL1, SATB2, TGFβ3, TGFβR1, TGFβR2, TP63, TWIST1, WNT3,* and *WNT5A*.

### Genomic loci likely associated to co-occurrence of orofacial clefting and tooth agenesis

In our literature search, some genomic loci, either deleted or containing mutations, were reported in patients with the co-occurrence of TA and OFCs. Schinzel and Schmid (1980) reported a patient with a deletion of 1q21–q25 [del(1)(q21–q25)] exhibiting OFC and TA (Table 4). This large deletion encompassed 702 genes, including protein-coding, non-coding genes, miRNAs and long non-coding RNAs (Supplementary Table 6).

A new ectodermal dysplasia-like syndrome, named del(2q32) syndrome, has been proposed in a case report of a patient with a 26 Mb interstitial deletion involving the region 2q31.2–q33.2, who showed multiple phenotypes including CP and TA, as well as severe intellectual disability and ectodermal anomalies (Rifai et al. 2010) (Table 4, Supplementary Table 6). Another 3.7 Mb deletion affecting the locus 4p16.3 was described by Maas et al. (2008) in a patient with Wolf–Hirschhorn syndrome (OMIM# 194190) exhibiting CP and TA along with intellectual disability, microcephaly at birth and other abnormalities (Table 4; Supplementary Table 6). Three studies reported on patients with oropalatal Bettex–Graf dysplasia, combining CP and HD or OD with a fragile site located in the region 16q22 (Table 4; Supplementary Table 6) (Bettex et al. 1998; Janiszewska-Olszowska et al. 2013; McKenzie et al. 2002).

Another locus associated with a known cleft syndrome, DiGeorge syndrome (OMIM# 188400), is located on chromosome 22 containing the main causative gene, *TBX1*. In four young patients with DiGeorge syndrome, the deletion of the locus 22q11 was diagnosed along with a CP and TA (Table 4; Supplementary Table 6) (Heliövaara et al. 2011).

A family-based study published in 2012 (Yildirim et al. 2012) confirmed the significant association between an intragenic SNP (rs987525) affecting a long non-coding RNA gene, LINC00976, located in the 8q24.21 region (Table 4; Supplementary Tables 4 and 6), and the co-occurrence of OFCs and TA. This evidence is particularly interesting since rs987525 has been demonstrated to be a susceptibility marker for non-syndromic CL/P in human as well as in animal models although the molecular mechanisms explaining the involvement of LINC00976 in OFC development remain unknown (Birnbaum et al. 2009; Mangold et al. 2011; Uslu et al. 2014). On the contrary, no studies have been published so far to validate a possible association between the 8q24 locus and TA, thus our hypothesis of a possible association represents a first input to stimulate molecular and functional studies in vitro and in vivo to shed light on potentially novel pathogenic mechanisms for TA and OFCs.

## Discussion

The aim of this systematic review is to thoroughly and systematically investigate the available literature to collect a panel of genes and loci likely contributing to the co-occurrence of TA and OFC in humans, possibly confirmed in animal models, and to speculate on the possible key pathways involved in physiological tooth development and in facial primordia migration, proliferation and fusion.

Although the co-occurrence of dental anomalies, specifically TA, and OFCs is frequently seen clinically, a comprehensive molecular and genetic exploration of the possible key genes for the common pathogenesis has not been performed so far. This is mainly explained by the fact that the mildest forms of TA are often neglected or overlooked compared to the more severe OFCs.

Overall, from this systematic literature search we identified 84 articles fulfilling our inclusion criteria. Based on them, 26 genes and 9 genomic loci emerged as related to the oral defects of interest (Tables 1, 2, 3; Supplementary Tables 4 and 6). Among the 26 genes, the majority belongs to known OFC- or TA-related pathways (Tables 1, 2, 3; Supplementary Table 4). Some of these genes encode transcription factors differentially involved in the regulation of embryonic developmental events, working synergistically in some cases: MSX1 and PAX9, CHD7, TWIST1, TP63 and IRF6, and the homeodomain proteins SATB2, TBX22 and PITX2. Other candidates encode for effectors of signaling pathways that lead to the modulation of cell differentiation, migration or adhesion. This is the case for CDH1 and PVRL1, the SHH receptor PTCH1, for TGFα, TGFβ3 and its receptors TGFβR1/2, for FGF8 and FGFR1, and for AXIN2, WNT3 and its regulator OFD1. In addition, we found two enzymes, KMT2D and KDM6, that specifically modify chromatin structure, thus regulating transcription by epigenetic modifications. Moreover, the DNA-binding repressor protein, BCOR, was also identified, acting by inhibiting gene expression when recruited to specific promoter regions.

To speculate on the possible clustering of these 26 genes (Tables 1, 2, 4; Supplementary Table 4), the GO terms indicating the biological processes which involve these genes have been collected and subsequently mapped to broader GO-slim categories. Based on these generic GO-slim categories, we proposed a gene super-clustering, including six partially overlapping super-clusters: (a) anatomical development, (b) cell division, growth and motility, (c) cell metabolism and catabolism, (d) cell transport and signal transduction, (e) cell structure organizations and (f) organ/system-specific processes (Supplementary Table 5). As we expected, 23 of the 26 candidate genes described in this review are implicated in embryogenesis, morphogenesis, anatomical patterning and maturation, as well as neural crest formation, further supporting the hypothesis about their involvement in tooth, lip and palate formation. In addition, 23 of the 26 candidate genes contribute to general cellular metabolism and catabolism as well as general cell processes, such as differentiation, proliferation and migration, while 21 are related to cell transport and signal transduction. Out of 26 candidate genes, 17 are involved in cell structure organization, like membrane and cytoskeleton formation. While, another group of 17 candidate genes has been associated with organ and system-specific processes, such as those which take place specifically in the nervous system and in the circulatory system. To discriminate which of the presented candidate genes have molecular functions that indeed underlie the failure of facial primordia migration and fusion, and the disruption of tooth development, and those whose functions could not explain their dual role in the pathogenesis of these conditions, further molecular analyses based on genotyping and population screening in concert with animal model studies and bioinformatics tools are necessary.

The fact that OFCs and TA are congenital birth defects starting to develop in the orofacial region of the 6–12-week human embryo largely explains why the GO term-based super-clustering (first super-cluster) is focusing on genes known to drive embryogenesis and oral morphogenesis. The clustering based on the GO terms in relation to biological processes have been used to identify common genes and pathways involved in TA and OFCs, and to predict possible new interactions between candidates in the same biological processes. However, since the clustering is a bioinformatics prediction, it will be of interest to validate the resulting clusters and to underpin the related hypotheses with functional studies.

Some of the presented genes, like *TP63* and *IRF6*, are well-known and widely studied genes, especially in relation to OFCs, and in our GO term analysis they are present in all the clusters, suggesting that those genes are governing broad molecular networks. The *TP63* gene plays a critical role in epithelial differentiation and in our gene set it represents the gene most likely contributing to the pathogenesis of both TA and OFCs as it takes part in the development and maintenance of stratified epithelial tissues, mediating the interactions between the mesenchyme and the epithelium. Mutations in *TP63* also underlie several dysmorphology syndromes including clefts or cleft features.

The *IRF6* gene is a target of *TP63* that activates *IRF6* transcription through the *IRF6* enhancer element (Dixon et al. 2011). *IRF6* is related to the formation of connective tissue (for example in the palate), the palatal rugae and underlying the dental epithelium (Blackburn et al. 2012; Chu et al. 2016). *IRF6* can be considered as the second gene most likely playing a role in the combined OFC–TA phenotype since it is the causative gene of van der Woude syndrome, whose typical features are OFCs, TA and lip pits.

Similarly, *MSX1* and *PAX9* have also been associated with the co-occurrence of OFCs and TA, as implicated in the development of cephalic structures and dental development both in humans and animal models. Interestingly, some of the candidate genes belong to five major gene families, WNTs, FGFs, BMPs, TGFs and PAXs, protein families that are essential in different phases of neural crest development, the structure from which originate the facial primordia, the palatal shelves, the alveolar ridge and the teeth. During the initial neural crest cell specification phase, the concerted activity of the WNT, FGF and BMP pathways induces the expression of neural plate border (or neural crest) genes, which turn on the expression of a distinct set of transcription factors including MSX1 and PAXs, and neural crest cells genes such as *TWIST1* and *Myc*, representing the neural crest specification module factors that determine the neural or non-neural fate of the neural crest cells (Simões-Costa and Bronner 2015).

In addition, a new level of gene expression regulation has been recently added to the cis-transcriptional regulation programs, i.e., the epigenetic regulation based on chromatin remodeling. For the neural crest specification, the removal of repressive methylation marks is necessary as well as the addition of acetylation marks to relax the chromatin structure making it more accessible to DNA-binding transcription factors. Interestingly, two of the presented candidate genes, *KMT2D* and *KDM6A* (a methyltransferase and a demethylase, respectively), regulate the chromatin methylation status and they are both causes of Kabuki syndrome, whose wide spectrum of features includes also OFCs and TA.

As the neural crest is one of the most conserved structures in vertebrates, the overlap of genes active during neural crest formation, odontogenesis, palatogenesis and facial primordia development may reflect the importance of the teeth and the palate, also in terms of human evolution and survival.

Although it would have been interesting to check for maternal genetic effects in these combined OFC–TA phenotypes, it was striking that in most articles relevant for our literature search the genotype of the mother was not provided. As our study almost exclusively focuses on inherited genetic effects, based on the genotypes of the described cases, we could not draw any conclusion on a possible implication of maternal genetic effects, which in other studies are acknowledged to affect the susceptibility of the embryo, and increasing the risk to develop a cleft. We, therefore, estimate that from the current stage of knowledge in the literature concerning the co-occurrence of OFC and TA, we are not able to provide any clues on the maternal genetic effects on the incidence or prevalence of these phenotypes.

As our review is a survey of scientific literature to identify genes involved in cases with co-occurrence of OFCs and TA, epidemiological analyses are beyond the specific aim of our study. We are aware indeed that some of the identified genes could be involved in OFC cases with TA by chance but, based on the sole data that is available in the literature, it seems impossible to track them down at this stage. However, the results of this study provide an intriguing list of candidate genes which could eventually be tested in prospective studies to sort out those that were not specifically associated with OFC–TA co-occurrence.

Nevertheless, with our review we aim to boost the research in this direction: we would like to draw the attention of clinicians and researchers working in TA and OFC field, on the investigation of the co-occurrence of these two defects, rather than focusing on the one or the other single oral defect. If the new combinatorial perspective will be adopted and developed in the following years, it would be easier in the future to get a concrete overview of the risk of TA–OFC co-occurrence in the population.

It would also be interesting to get a sense of how much of risk for each defect is attributable to recognized genetic loci, but our review is neither a meta-analysis nor an epidemiologic study. Presently, the number of patients reported in the literature is far too low for most of the genes, so that a sturdy statistical analysis is not possible. Therefore, we decided to report the number of patients found for each locus/gene (Tables 1, 2, 4) to give the reader a sense of how rarely the TA–OFC co-occurrence develops in case of gene/locus mutations. This way we avoided to perform a statistical analysis that would never have been unbiased and reliable in the present situation.

It is too early for translating the review findings into therapeutic strategies for congenital birth defects like orofacial clefts with (*or without*) tooth agenesis, as these birth defects are different from other genetic diseases like retinitis pigmentosa (Bassuk et al. 2016) and Duchenne’s muscular dystrophy (Wojtal et al. 2016) for which postnatal treatments with genetic editing in patient-derived stem cells are currently under development. However, the identified OFC–TA genes could eventually be included in postnatal diagnostic gene panels. Advantages of postnatal genetic testing not only includes more precise (sub)phenotyping of the patient, but also targeted physical examination of the patients’ parents as well as members of his/her broader family. The latter can also be approached for genotyping and for deep phenotyping, including OFC and TA (sub)phenotypes. In fact, in children born with apparently isolated OFC, an NGS-based screening of a panel of genes including both syndromic and TA-OFC genes could be useful for the diagnosis of cleft syndromes and tooth agenesis, and lead to earlier onset of therapies. However, the diagnosis of a case with OFC (*with or without TA*) but without other severe malformations does not influence future pregnancy planning as these conditions are treatable. In next pregnancies, testing OFC–TA genes could eventually be carried out on amniocytes to anticipate diagnosis. For these cases, however, termination of pregnancy should never be an option offered by clinical geneticists.

In addition, the exact role of these genes, loci and pathways in orofacial development should first be further elucidated with functional in vitro and in vivo studies to increase our understanding of the molecular mechanisms that lead to orofacial clefts in case of disruption. Tooth development, palatal shelf migration and lip formation, although depending on different tissues, timing and dynamics, are based on similar processes including cell migration and fusion. It is not fully clear if the same genes drive these processes in different tissues, but the co-occurrence of TA and OFCs due to the disruption of specific genes may support this hypothesis.

Intriguingly, not only the disrupted gene but even the location of the mutations within the gene can lead to diverse phenotypes. A recently published review (Liang et al. 2016) shows how the location of mutations in the MSX1 homeodomain always causes TA with or without other phenotypes while mutations outside the homeodomain are mostly associated with non-syndromic OFCs. Following this hypothesis, it would be interesting for further functional studies to expand the molecular investigation to different protein domains in relation to different spectra of phenotypes, thus improving the diagnostic potential of these gene panels and the knowledge of molecular pathogenic mechanisms affecting the orofacial region.

## Electronic supplementary material

Below is the link to the electronic supplementary material.

Supplementary Table 1 Literature searches: histories and search terms. (DOCX 24 kb)

Supplementary Table 2 All articles resulting from the literature search. (XLSX 46 kb)

Supplementary Table 3 Articles included based on the contents. (XLSX 19 kb)

Supplementary Table 4 Human mutations described as affecting the candidate genes reported in the included articles. (XLSX 28 kb)

Supplementary Table 5 Slim GO term analysis and gene clustering. (XLSX 23 kb)

Supplementary Table 6 Genomic loci identified from the included articles and their encompassed genes. (XLSX 39 kb)
