# Supplementary material for: Tooth agenesis and orofacial clefting: genetic brothers in arms?
Source: Hum Genet. 2016 Oct 3;135(12):1299–327. doi: 10.1007/s00439-016-1733-z (PMC5065589; doi:10.1007/s00439-016-1733-z)
Supplement: Supplementary file 1 — Literature searches: histories and search terms. (DOCX 24 kb) [file 439_2016_1733_MOESM1_ESM.docx]

***Supplementary Table 1. Literature searches: histories and search terms.***

EMBASE search history

| **No.** | **Search query** | **Results** |
| --- | --- | --- |
| #1 | cleft lip/ or cleft lip face palate/ or cleft lip nose/ or cleft lip palate/ or unilateral cleft lip/ or van der woude syndrome/ | 14804 |
| #2 | palate malformation/ or cleft lip face palate/ or cleft palate/ or roberts syndrome/ or van der woude syndrome/ or velocardiofacial syndrome/ | 20544 |
| #3 | face malformation/ or cleft face/ or craniofacial malformation/ or dentofacial deformity/ | 11529 |
| #4 | (orofacial cleft or orofacial clefts or mandible cleft or mandible clefts or mandibular cleft or mandibular clefts or cleft lip or lip clefts or cleft palate or palate clefts or facial cleft or facial clefts or face cleft or face clefts or alveolar ridge cleft or alveolar ridge clefts or dental ridge cleft or dental ridge clefts or cleft alveolus).tw. | 18569 |
| #5 | #1 or #2 or #3 or #4 | 37786 |
| #6 | chromosome mutation/ or gene mutation/ or germline mutation/ or somatic mutation/ or spontaneous mutation/ | 297093 |
| #7 | exp genetic predisposition/ | 88585 |
| #8 | exp gene/ | 742484 |
| #9 | (mutation or mutations or polymorphism or polymorphisms or SNP or SNPs or genetic association study or genetic association studies or GWA or GWAS or gene or genes or locus or loci or animal model or animal models or mouse model or mouse models or zebrafish model or zebrafish models).tw. | 2394248 |
| #10 | 6 or 7 or 8 or 9 | 2605257 |
| #11 | hypodontia/ or oculodentodigital syndrome/ or oligodontia/ or van der woude syndrome/ | 3882 |
| #12 | hypodontia/cn, et [Congenital Disorder, Etiology] | 443 |
| #13 | (oligodontia or anodontia or hypodontia or tooth agenesis or dental agenesis).tw. | 2099 |
| #14 | 11 or 12 or 13 | 4294 |
| #15 | 5 and 10 | 5985 |
| #16 | 14 and 10 | 991 |
| #17 | 15 and 16 | **281** |

Legend: orofacial clefts, yellow; ano-/oligo-/hypodontia, blue; genes/genetics terms, orange; combined topics, green.

Notes: no subheadings available for the terms *mandibular cleft* and *alveolar ridge cleft*.

PubMed search history

| [**No.**](http://ovidsp.tx.ovid.com.proxy.ubn.ru.nl/sp-3.14.0b/ovidweb.cgi?&S=GLNHFPBNDKDDLDKHNCLKAAGCHJBBAA00&Sort+Sets=descending) | **Search query** | **Results** |
| --- | --- | --- |
| #1 | Search (((((((((("Cleft Lip"[Mesh]) OR "Acrofrontofacionasal dysostosis syndrome" [Supplementary Concept]) OR "Ladda Zonana Ramer syndrome" [Supplementary Concept]) OR "Contractures ectodermal dysplasia cleft lip palate" [Supplementary Concept]) OR "Kallmann Syndrome"[Mesh]) OR "Ectrodactyly and Ectodermal Dysplasia without Cleft Lip/Palate" [Supplementary Concept]) OR "Juberg Hayward syndrome" [Supplementary Concept]) OR "Leichtman Wood Rohn syndrome" [Supplementary Concept]) OR "Orofaciodigital syndrome 6" [Supplementary Concept]) OR "Crane-Heise syndrome" [Supplementary Concept]) OR "Roberts Syndrome" [Supplementary Concept] | 12652 |
| #2 | Search ((((((("Cleft Palate"[Mesh]) OR "Jones Hersh Yusk syndrome" [Supplementary Concept]) OR "Kallmann Syndrome"[Mesh]) OR "Pierre Robin Syndrome"[Mesh]) OR "Brain Anomalies, Retardation, Ectodermal Dysplasia, Skeletal Malformations, Hirschsprung Disease, Ear/Eye Anomalies, Cleft Palate/Cryptorchidism, And Kidney Dysplasia/Hypoplasia" [Supplementary Concept]) OR "Cleft Palate-Lateral Synechia Syndrome" [Supplementary Concept]) OR "Growth and mental retardation, mandibulofacial dysostosis, microcephaly, and cleft palate" [Supplementary Concept]) OR "Radial defect Robin sequence" [Supplementary Concept] | 18411 |
| #3 | Search ((((((((((("Microcephaly, corpus callosum dysgenesis and cleft lip-palate" [Supplementary Concept]) OR "Schrander-Stumpel Theunissen Hulsmans syndrome" [Supplementary Concept]) OR "Orofaciodigital syndrome 6" [Supplementary Concept]) OR "Orofaciodigital syndrome 5" [Supplementary Concept]) OR "Juberg Hayward syndrome" [Supplementary Concept]) OR "Kapur Toriello syndrome" [Supplementary Concept]) OR "Yim Ebbin syndrome" [Supplementary Concept]) OR "Median cleft lip, corpus callosum, lipoma, and skin polyps" [Supplementary Concept]) OR "Roberts Syndrome" [Supplementary Concept]) OR "Facial Dysmorphism, Cleft Palate, Hearing Loss, and Camptodactyly" [Supplementary Concept]) OR "Frontonasal dysplasia" [Supplementary Concept]) OR "Leichtman Wood Rohn syndrome" [Supplementary Concept] | 53 |
| #4 | Search "Richieri Costa Pereira syndrome" [Supplementary Concept] | 6 |
| #5 | Search (((((((((((("Orofacial Cleft 1" [Supplementary Concept]) OR "Orofacial Cleft 12" [Supplementary Concept]) OR "Orofacial Cleft 11" [Supplementary Concept]) OR "Orofacial Cleft 10" [Supplementary Concept]) OR "Orofacial Cleft 2" [Supplementary Concept]) OR "Orofacial Cleft 4" [Supplementary Concept]) OR "Orofacial Cleft 5" [Supplementary Concept]) OR "Orofacial Cleft 9" [Supplementary Concept]) OR "Orofacial Cleft 3" [Supplementary Concept]) OR "Hypodontia Oligodontia with Orofacial Cleft" [Supplementary Concept]) OR "Tooth Agenesis, Selective, With Orofacial Cleft" [Supplementary Concept]) OR "Orofacial Cleft 8" [Supplementary Concept]) OR "Orofacial Cleft 7" [Supplementary Concept] | 27 |
| #6 | Search (cleft lip[tiab] OR lip cleft[tiab] OR lip clefts[tiab]) | 10465 |
| #7 | Search (cleft palate[tiab] OR palate cleft[tiab] OR palate clefts[tiab]) | 10823 |
| #8 | Search (facial cleft[tiab] OR facial clefts[tiab] OR face cleft[tiab] OR face clefts[tiab]) | 4491 |
| #9 | Search (mandible cleft[tiab] OR mandible clefts[tiab] OR mandibular cleft[tiab] OR mandibular clefts[tiab]) | 1035 |
| #10 | Search (alveolar ridge cleft[tiab] OR alveolar ridge clefts[tiab] OR dental ridge cleft[tiab] OR dental ridge clefts[tiab] OR alveolar cleft[tiab] OR alveolar clefts[tiab] OR cleft alveolus[tiab]) | 847 |
| #11 | Search (orofacial cleft[tiab] OR orofacial clefts[tiab]) | 710 |
| #12 | Search (#1 OR #2 OR #3 OR #4 OR #5 OR #6 OR #7 OR #8 OR #9 OR #10 OR #11) | 26823 |
| #13 | Search (((((((((((("Regulatory Sequences, Nucleic Acid"[Mesh]) OR "Polymorphism, Genetic"[Mesh]) OR "Genetic Association Studies"[Mesh]) OR "Genetic Loci"[Mesh]) OR "Genetic Research"[Mesh]) OR "Genetic Diseases, Inborn"[Mesh]) OR "Genetic Predisposition to Disease"[Mesh]) OR "Genetic Variation"[Mesh]) OR "Skin Diseases, Genetic"[Mesh]) OR "Genetic Linkage"[Mesh]) OR "Genes, Recessive"[Mesh]) OR "Genes, Dominant"[Mesh]) OR "Genes"[Mesh] | 1731919 |
| #14 | Search (mutation[tiab] OR mutations[tiab] OR polymorphism[tiab] OR polymorphisms[tiab] OR SNP[tiab] OR SNPs[tiab] OR genetic association study[tiab] OR genetic association studies[tiab] OR GWA[tiab] or GWAS[tiab] OR gene[tiab] OR genes[tiab] OR locus[tiab] OR loci[tiab] OR animal model[tiab] OR animal models[tiab] OR mouse model[tiab] OR mouse models[tiab] OR zebrafish model[tiab] OR zibrafish models mouse model[tiab]) | 2051543 |
| #15 | Search (#13 OR #14) | [2836433](http://www.ncbi.nlm.nih.gov/pubmed/?cmd=HistorySearch&querykey=143) |
| #16 | Search "Anodontia"[Mesh] | 3112 |
| #17 | Search ((((((((("Deafness oligodontia syndrome" [Supplementary Concept]) OR "Tooth Agenesis, Selective, X-Linked, 1" [Supplementary Concept]) OR "Tooth Agenesis, Selective, 3" [Supplementary Concept]) OR "Hypodontia Oligodontia with Orofacial Cleft" [Supplementary Concept]) OR "Tooth Agenesis, Selective, 2" [Supplementary Concept]) OR "Cleft Palate, Deafness, and Oligodontia" [Supplementary Concept]) OR "Tooth Agenesis, Selective, 5" [Supplementary Concept]) OR "Leukodystrophy, Dysmyelinating, with Oligodontia" [Supplementary Concept]) OR "Oligodontia-Colorectal Cancer Syndrome" [Supplementary Concept]) OR "Martinez Monasterio Pinheiro syndrome" [Supplementary Concept] | 2 |
| #18 | Search ((((((((((((((("Hypodontia, X-linked" [Supplementary Concept]) OR "Witkop syndrome" [Supplementary Concept]) OR "Leukodystrophy, Hypomyelinating, with Hypodontia and Hypogonadotropic Hypogonadism" [Supplementary Concept]) OR "Tooth Agenesis, Selective, X-Linked, 1" [Supplementary Concept]) OR "Tooth Agenesis, Selective, 3" [Supplementary Concept]) OR "Hypodontia Oligodontia with Orofacial Cleft" [Supplementary Concept]) OR "Split-Hand And Split-Foot With Hypodontia" [Supplementary Concept]) OR "Tooth Agenesis, Selective, 2" [Supplementary Concept]) OR "Tooth Agenesis, Selective, 5" [Supplementary Concept]) OR "Schopf-Schulz-Passarge Syndrome" [Supplementary Concept]) OR "Leukomelanoderma, Infantilism, Mental Retardation, Hypodontia, Hypotrichosis" [Supplementary Concept]) OR "Propping Zerres syndrome" [Supplementary Concept]) OR "Deafness oligodontia syndrome" [Supplementary Concept]) OR "Microdontia hypodontia short stature" [Supplementary Concept]) OR "Zadik Barak Levin syndrome" [Supplementary Concept]) OR "Mehta Lewis Patton syndrome" [Supplementary Concept] | 16 |
| #19 | Search ((((((((("Tooth Agenesis, Selective, 4" [Supplementary Concept]) OR "Tooth Agenesis, Selective, 6" [Supplementary Concept]) OR "Tooth Agenesis, Selective, X-Linked, 1" [Supplementary Concept]) OR "Facial Dysmorphism, Selective Tooth Agenesis, and Choroid Calcification" [Supplementary Concept]) OR "Tooth Agenesis, Selective, 3" [Supplementary Concept]) OR "Tooth Agenesis, Selective, With Orofacial Cleft" [Supplementary Concept]) OR "Kallmann Syndrome 2 with Selective Tooth Agenesis" [Supplementary Concept]) OR "Tooth Agenesis, Selective, 2" [Supplementary Concept]) OR "Tooth Agenesis, Selective, 5" [Supplementary Concept]) OR "Oligodontia-Colorectal Cancer Syndrome" [Supplementary Concept] | 2 |
| #20 | Search oligodontia[tiab] OR anodontia[tiab] OR hypodontia[tiab] OR tooth agenesis[tiab] OR dental agenesis[tiab] | 2161 |
| #21 | Search (#16 OR #17 OR #18 OR #19 OR #20) | 3947 |
| #22 | Search (#12 AND #15) | 5304 |
| #23 | Search (#21 AND #15) | 1343 |
| #24 | Search (#23 AND #24) | **166** |

Legend: orofacial clefts, yellow; ano-/oligo-/hypodontia, blue; genes/genetics terms, orange; combined topics, green.
